# Unmet need for modern contraceptives and associated factors among women in the extended postpartum period in Dessie town, Ethiopia

**DOI:** 10.1186/s40834-017-0048-3

**Published:** 2017-08-07

**Authors:** Masresha Tegegn, Mastewal Arefaynie, Tenaw Yimer Tiruye

**Affiliations:** 1 Adolescent reproductive health service officer, family guidance association of Ethiopia (FGAE), south area office, Dessie, Ethiopia; 2 Public health department, college of medicine and health sciences, Wollo University, Dessie, Ethiopia; 3 Public health department, college of health sciences, Debre Markos University, PO Box: 269, Debre Markos, Ethiopia

**Keywords:** Unmet need, Modern contraceptives, Associated factors, Extended postpartum period, Dessie town, North east Ethiopia

## Abstract

**Background:**

The contraceptive use of women in the extended postpartum period is usually different from other times in a woman’s life cycle due to the additional roles and presence of emotional changes. However, there is lack of evidence regarding women contraceptive need during this period and the extent they met their need. Therefore, the objective of this study was to assess unmet need for modern contraceptives and associated factors among women during the extended postpartum period in Dessie Town, North east Ethiopia in December 2014.

**Methods:**

A community-based cross-sectional study was conducted among women who gave birth one year before the study period. Systematic random sampling technique was employed to recruit a total of 383 study participants. For data collection, a structured and pretested standard questionnaire was used. Descriptive statistics were done to characterize the study population using different variables. Bivariate and multiple logistic regression models were fitted to control confounding factors. Odds ratios with 95% confidence intervals were computed to identify factors associated with unmet need.

**Results:**

This study revealed that 44% of the extended post-partum women had unmet need of modern contraceptives of which 57% unmet need for spacing and 43% for limiting. Education of women (being illiterate) (AOR (adjusted odds ratio) =3.37, 95% CI (confidence interval) 1.22–7.57), antenatal care service (no) (AOR = 2.41, 95% CI 1.11–5.79), Post-natal care service (no) (AOR = 3.63, CI 2.13–6.19) and knowledge of lactational amenorrhea method (AOR = 7.84 95% CI 4.10–15.02) were the factors positively associated with unmet need modern contraceptives in the extended postpartum period.

**Conclusion:**

The unmet need for modern contraception is high in the study area. There is need to improve the quality of maternal health service, girls education, information on postpartum risk of pregnancy on the recommended postpartum contraceptives to enable mothers make informed choices of contraceptives.

## Background

The measure of unmet need for contraception represents a core concept in the field of family planning and one of the most important indicators for family planning policy, programs and research [1–5].

Few studies have assessed the value of the unmet need measure for predicting future contraceptive use or compared outcomes among women classified as having an unmet need according to whether or not the women intend to use a method [6–9].

In Ethiopia, where the total fertility rate was 4.8 children per women and contraceptive prevalence rate was only 29%, unmet need for family planning was 25%. This Figure in the postpartum period was different, only 20% of contraceptive prevalence and the unmet need increase to 76% [10]. Extended postpartum period is defined as a time between birth and one year, which is important time to prevent child as well as maternal mortality and morbidity by reducing high risk pregnancy (11). Hence, concentrating efforts to reduce unmet need among women during this critical period has a bigger impact on increasing contraceptive use than concentrating on any other group [4, 11–19].

While extensive literatures were available on the associated factors of unmet need for contraceptives among general reproductive age women [10, 20–22], there is lack of evidence among women in the extended postpartum period. Therefore, the objective of this study was to assess unmet need for contraceptives among women during the extended postpartum period in Dessie North East Ethiopia.

## Methods

The study was conducted in Dessie town, located 400 km from North east of Addis Ababa, capital of Ethiopia. It is divided to 10 urban and 6 rural Kebeles (lowest administrative unit in Ethiopia). According to the 2014 Dessie town health office estimate, there were 195,661 residents in the town and 52% of them were females. Using the conversion factor of 3.37% to estimate the number of women having children less than one years old, the estimated number of postpartum women were 6594 [23]. The town had four hospitals and eight health centers providing maternal and other health services to the population.

A quantitative cross sectional community based study design was conducted during December 2014 among 383 systematically selected women who gave birth one year before the study period. The sample size for first specific objective was calculated using a single population proportion formula with the following assumptions: a 95% confidence interval (Z value of 1.96), 5% marginal error and 86% proportion of unmet need in the extended post-partum period (taken from study in rural Uganda) [24]. The final sample size determined by the formula n = (Z_ɑ⁄2_)^2^ P (1-P) ⁄d^2^ with an assumption of 10% non–response rate that gives 383. Sample for second specific objective was calculated by double proportion formula using EPI info 95% CI and 90% power R1 for exposed & R2 for non-exposed that give total sample size of 112. Thus, larger sample size, which was calculated for first objective 383, was taken.

To select study subjects, first all the sixteen (10 urban and 6 rural) Kebeles were listed with their population size. The sampling frame prepared from Health workers registration document for immunization purpose. Then, the number of sample divided for each Kebele based on proportional allocation for size (Table 1). Then, households selected the sample every K^th^ interval by systematic random sampling technique. Finally, women in each selected house that met inclusion criteria were interviewed.

**Table 1:** Sampling size determination (proportional allocation to size), Dessie town, northeast Ethiopia, 2014 (*n* = 383)

Kebele	Total population	Total no of surviving infant under one year age = (Total population in the Kebele) X (3.37%)	Sample size taken from each Kebele =Total under one in the Kebele X 383 Total infants in the town
1.	13,415	424	26
2.	13,853	438	27
3.	24,429	772	48
4.	16,105	509	32
5.	14,464	457	28
6.	19,360	612	38
7.	10,027	317	20
8.	16,568	524	32
9.	9903	313	19
10.	23,018	727	45
11.	5340	169	10
12.	7922	250	16
13.	3485	110	7
14.	6302	199	12
15.	7154	226	14
16.	4317	136	8
Total	195,661	6180	383

From rural kebeles (with total population of 35, 219) = 68 and from urban kebeles (with total population of 160, 442) = 315

The dependent variable was unmet need for modern contraceptives during extended post partum period (Women not use modern contraceptives but want to space or limit = Yes, and women utilize modern contraceptives and women who wants to give birth soon = No and) that was obtained prospectively as it is more likely to correlate with the need for family planning during the postpartum period (taken as the first one year postpartum for this analysis). The independent variables were: socioeconomic and demographic factors (age, education, religion, ethnicity, marital status, living arrangement, number of living children, wealth quintile, decision making autonomy, spousal communication and occupation), maternal health service factors (ANC, PNC, Site of Delivery and FP counseling), contraceptive knowledge, service availability and source of information an Pregnancy risk perception.

In this study: *Extended Postpartum family planning* refers to initiation and use of family planning methods during the first years after giving birth, *unmet need* of contraception implies all women in extended post-partum period who not currently using modern contraceptive method and want to limit or post pone their next birth at least for two year, *postpartum amenorrhea* is the interval between the birth of a child and the resumption of menses and *modern contraceptives* implies methods including sterilization, pills, intrauterine contraceptive device (IUCD), injectable, implants and condom. Women have contraception knowledge if they list at least one modern contraceptive.

Data collection questionnaire was adapted from Ethiopian demographic and health survey, 2011 (12). The tool was structured and translated to local Amharic language. The data was collected by trained and experienced data collectors and supervised by BSc nurses. The tool was pretested on 20 participants. Daily supervision of the data collection was done and data were randomly checked. All the information which was collected from the household were asked by data collectors and checked. The questionnaire was checked for completeness on daily basis.

The collected data were double entered using EPI-INFO version 7 and exported to SPSS version 16.0 for data processing and analysis. Descriptive tests like proportions mean and standard deviations and analytic tests like bivariate and multivariate logistic regression analysis were computed. Odds ratio along with the 95% CI was estimated to ascertain the association between covariates and unmet need of contraception the extended post partum period. Covariates that have *P*-value of <0.25 at the bivariate analysis were included in the multivariate logistic regression to control all possible confounding factors. For all statistical tests *P*-value ≤0.05 was used as a cut-off point for statistical significance.

Ethical clearance was obtained from the ethical clearance committee of Wollo University, College of Medicine and Health Science. Official consent letter was issued from Dessie town administrative health Office. The necessary explanation about the purpose of the study and about its procedure was done and verbal consent was obtained from each respondent. To assure the confidentiality of the response, anonymous interview was conducted.

## Results

### Socio demographic characteristics of study subjects

From a total of 383 women who were in the first year after delivery, 382 were interviewed and its response rate was 99.7%. Sixty nine (18%) of the study subjects were living in rural area of the town. Two hundred twenty five (58.9%) were age between 25 and 34 years. The mean age of the study subjects were 28 years with standard deviation of 5.48 (SD ± 5.48). Two hundred ninety eight (78%) of the respondents were married, 323 (84.6%) were Amhara by Ethnicity and 46.6% were Muslims. Two hundred fifteen (56%) were housewives. One hundred forty seven (38.5%) were attended primary and one hundred fifty seven (47.9%) secondary and above school (Table 2).

**Table 2:** Socio demographic characteristics of study subjects, Dessie town, northeast Ethiopia, 2014 (*n* = 382)

Variable	Number	Percentage	Unmet need	
			Yes	No
Age Group	No	%	No	No
15–24	98	29.7	59	39
25–34	225	58.9	91	134
35+	59	15.4	23	36
Residence				
Urban	313	82	137	176
Rural	69	18	36	33
Current Marital status				
Married	339	88.7	151	188
Separated/Divorced/Widowed	25	6.5	11	14
Single	18	4.7	11	7
Educational level				
Non educated	52	13.6	37	15
Primary	147	38.5	66	81
Secondary/Tertiary	183	47.9	70	113
Religion				
Orthodox	172	44.8	76	86
Protestant	33	8.6	15	18
Muslims	187	46.6	82	105
Ethnicity				
Amhara	324	84.6	145	179
Tigre	55	13.4	28	27

### Maternal health related characteristics of study subjects

#### Reproductive history

The average number of currently alive children was 2.22 per woman. One hundred fifty three (40%) had only one children. Seventy (18.3%) did not have a desire to have additional children. One hundred forty three (37.4%) of the respondents were reported that the current birth unintended and while 30 (7.9%) of the respondents did not want the current birth at all. More than half (51.3%) had not their menses resumed. Three hundred sixty six (95.8%) of them had resumed sexual intercourse. One hundred seventeen (30.6%) of the respondents were in between 13 and 26 weeks of post-partum period. Three hundred seventy five (98.2%) of the respondents were carry out breast feeding currently and 146(38.2%) had non-exclusive breast feeding.

#### ANC, PNC and delivery services

Three hundred twenty nine (86%) had ANC attendance. From the ANC attendants, 143 (43.3%) had attend four or more visits. Among the ANC attendants more than half (52.5%) were given counseling on modern contraceptives. Three hundred five (80%) of the respondents were delivered in health facilities. One hundred eight seven (49%) had a contact of family planning provider after delivery and received modern contraceptive counseling (Table 3).

**Table 3:** Reproductive health and maternal health service related characteristics of study participants, Dessie town, northeast Ethiopia, 2014 (*n* = 382)

Variable	Number	Percentage	Unmet need	
			Yes	No
No of Living Children				
1	153	40	67	86
2–3	170	44.5	72	98
≥ 4	59	15.4	34	25
Birth Interval in month (*n* = 305)				
< 2 years	34	11	12	22
2–3 years	189	62	105	84
4–5 years	63	21	50	13
More than five years	19	6	6	13
Menses return				
Yes	179	46.9	106	73
No	203	51.3	67	136
ANC follow up				
Yes	305	79.8	116	189
No	77	20.2	57	20
FP counseling during Pregnancy Period				
Yes	200	52.5	106	94
No	182	47.5	67	115
Contact of HP after Delivery/1st 45 days/				
Yes	187	49.	43	144
No	195	51	130	65

#### Family planning knowledge

Extended post-partum mothers were asked to name ways of preventing or delaying pregnancy. It was found that 352 (92%) mothers knew at least three modern contraceptive method, 341(89.3%) utmost two and only 15(4%) don’t know any modern contraceptives. Women were asked whether they had heard about any modern contraceptives before the current pregnancy and 367(96%) heard at least one method, 364(95%) had knew at utmost two places and 322(84%) knew at least three places. Women were asked whether they had used a contraceptive two years before the current pregnancy, more than two third of the respondents 239(61.8%) had used family planning while only 146 (38.2%) of the extended post-partum women reported not having used contraceptives before two years of the current birth. Three hundred five (82%) of the respondents had discussed on modern contraceptives with their partners. One hundred twenty seven (61%) of the respondents got the FP information from Health extension workers, seventy four (35%) from partners/relatives and the rest from radio and TV (Table 4).

**Table 4:** Distribution of Contraceptive knowledge of women in the extended post-partum period, Dessie town, northeast Ethiopia, 2014 (*n* = 382)

Variables	Number	Percent	Unmet need	
			Yes	No
Knowledge of modern contraceptives				
Don’t know	5	1.3	3	2
Utmost two	277	72.5	115	162
At least three	100	26.2	48	52
Ever heard about modern contraceptives				
Yes	367	96	160	207
No	15	4	13	2
Knowledge on Places of FP services provided				
Don’t know	5	1.3	3	2
Utmost two	275	72	118	157
At least three	102	26.7	40	62
Discussion with spouse on modern contraceptives				
Yes	305	82	133	172
No	69	18	40	19
Ever contraceptive use				
Yes	236	61.8	90	146
No	146	38.2	83	63
Current use of contraceptives				
Yes	199	52.0	0	209
No	183	48.0	173	10
Lactational amenorrhea knowledge				
Yes	204	53.4	51	153
No	178	46.6	122	56

#### Contraceptive use and reasons for non-use in the post-partum period

**Fig. 1:**
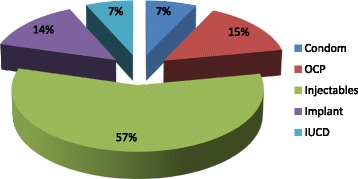
Modern contraceptive method mix in the extended post-partum period, Dessie town, northeast Ethiopia, 2014 (*n* = 382)

The prevalence of contraceptive use was found to be 209 (54.7%). Ninety four (45%) of the respondents got modern contraceptive from Government health facility. Injectable contraceptive, 120 (57.4%) and oral contraceptive pills 31(15%), Implant 29(14%), Condom 15(7.2%), IUCD 14 (6.7%) were use during the study period. Only Sixteen (7.7%) of the respondents had started to use family planning methods within the first three months (12 weeks) of post-partum period, 67(32%) started in the first 6 months after delivery; 153(73%) had started within the first nine months of post-partum period, while the reaming 56 (26.8%) had started after 9 month of post-partum period. Among those who had utilized post-partum contraceptives, 166 (60.3%) initiated contraceptives use after menses had resumed (Fig. 1).

**Fig. 2:**
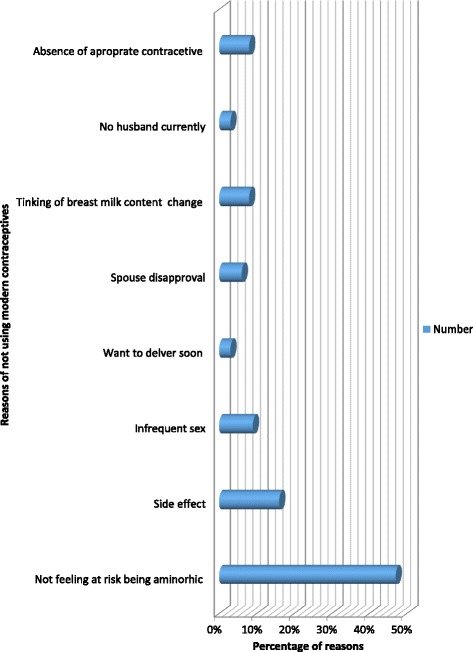
Reasons not use contraceptives among women in the extended post-partum period, Dessie town, northeast Ethiopia, 2014 (*n* = 173)

Among reasons of not using modern contraceptives in the extended postpartum period less risk perceived due to Amenorrhea (47%), fear of side effect (16%), infrequent sex (9%) and absence of preferred contraceptives (8%) were the most common (Fig. 2).

### Unmet need of modern contraceptives

**Fig. 3:**
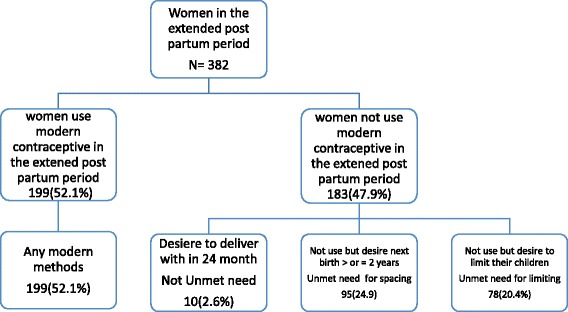
Unmet need of modern contraception among women in the extended post-partum period, Dessie town, northeast Ethiopia, 2014 (*n* = 382)

**I**n this study, 173 (45.2%) of the respondents had unmet need of modern contraceptives in the extended post-partum period. Among those who had unmet need of modern contraceptives ninety five (54.9%) had an unmet need for spacing and 78 (45.1%) for liming of children (Fig. 3).

### Associated factors of unmet need of modern contraceptives in the extended post-partum period

The results of bivariate and multivariate analysis between unmet need and selected independent factors are presented in Table 5. Accordingly, the following factors were independently associated with unmet need for modern contraception in the final multiple variable logistic regression model.

**Table 5:** Factors associated with unmet contraceptive need during extended postpartum period, Dessie town, northeast Ethiopia, 2014 (*n* = 382)

Variable	Unmet need		COR (95% CI)	AOR (95% CI)	*P*-value
	Yes	No			
Age					
≤ 24	59	39	1		
25–34	91	134	.45(.23, .73)	.744(.22, 2.52)	0.635
35+	23	36	.42(.22, .82)	.71(.25,1.99)	0.511
Women education					
Illiterate	37	15	3.52 (2.23, 4.88)	3.37(1.22, 7.57)^a^	0.017
Educated	136	194	1		
Living arrangement					
Alone/Parents	51	32	2.31(1.24, 4.89)	1.41(.15, 2.08)	0.071
Spouse	122	177	1	1	
Ever use of contraception					
Yes	90	146	1		
No	83	63	2.14(1.40, 3.25)	2.00(.90, 2. 82)	0.109
ANC service					
Yes	130	198	1		
No	43	11	5.95(2.96, 11.97)	2.41(1.11, 5.79)^a^	0.050
Site of delivery					
Health inst^n^	116	189	1		
Home	57	20	3.28(2.86, 5.90)	1.55(.59, 1.86)	0.071
Continuum of PNC					
Yes	43	144	1		
No	130	65	6.70(4.26, 10.53)	3.63(2.13, 6.19)^a^	0.0001
knowledge of lactational amenorrhea method					
No	51	153	1		
Yes	122	56	6.54(4.18, 10.23)	7.84(4.10, 15.02)^a^	0.001

^a^= significantly associated factors

Respondents who were illiterate were 3 and half times more likely to have unmet need of modern contraceptives in the extended post-partum period (AOR = 3.37, 95% CI 1.22–7.57) than women who are educated. On the other hand, women who had no ANC follow up before the current birth were 2.4 times more likely to have unmet need than who had ANC follow up (AOR = 2.41, 95% CI 1.11–5.79). Those mothers who were not received PNC were 3.6 times more likely to have unmet need (AOR = 3.63, CI 2.13–6.19). Mothers who are knowledgeable about lactational amenorrhea method about 8 times more likely to have unmet need (AOR = 7.84 95% CI 4.10–15.02) than their counterparts.

The other variables like religion, age of mother, occupation, wealth quintile, living arrangement, ever use of contraception, contraceptive knowledge and site of delivery were not statistically significant with unmet need of modern contraceptives among women the extended postpartum period.

## Discussion

In the present study, the prevalence of the unmet need for modern contraceptives was 44% which is much higher than compared to 2011 EDHS all married women of reproductive age (25%) [10] and studies from Nigeria, Nepal and Bangladesh [19, 25, 26]. The possible explanation for the observed difference is women in study area had recently given birth and may not have accessed family planning by the time of the survey. The other possible explanation for the observed variation in the prevalence could be due to the definition of unmet need, research design and screening instruments used that varied between settings, cultures and populations. DHS used standard instrument but it may suffer from recall bias as all reproductive age women are asked about their FP related experiences. Though this study used the DHS standard instrument, women were asked during their experiences in the first year after delivery (recent) to avoid this recall bias.

In this study, the association between variables and unmet need was assessed. Educational attainment of extended post-partum mothers is a major factor influencing to use postpartum contraception [27, 28]. Illiterate mothers were more likely to have unmet need than their counter parts. This is could be educated mothers are likely to be aware of unintended pregnancy (due to non-use of contraception) and its consequences, likely to marry educated husband that facilitate couples discussion on maternal health care utilization including FP, likely to be autonomous in decision making and hence meting her FP when she want.

ANC service was another factor that significantly affecting unmet need as women who attend antenatal care were less likely to have unmet need which is consistent with other studies [19, 20, 29]. This may be prenatal family planning counseling is one of the objectives included in the focused antenatal care strategy in Ethiopia [20–22]. Hence, it creates golden opportunities to get information towards contraceptive use, for post-partum FP discussion and education due to increased provider-patient interaction. This is also revealed from prospective study done in African countries [29]. Studies in Mexico have also shown that FP counseling during prenatal care would motivate women to practice contraceptives [30].

A number of studies have looked at the effect of post-partum FP contacts across a continuum of care, including prenatal care through postpartum and later care. This study revealed that women who were not received PNC were more likely to have unmet need. This is explained due to the fact that postnatal visit may give the opportunity for contraceptive counseling and FP adoption in the postpartum period [20, 22].

This study also revealed that mothers who are knowledgeable about lactational amenorrhea method (LAM) were more likely to have unmet need than their counterparts. Similar finding was reported from a study done in Nigeria and Kenya [19, 28]. This might be explained by the fact that amenorrhic women would underestimate the risk of pregnancy by assuming that amenorrhea could guarantee protection against pregnancy regardless of the time of postpartum period [31]. With this regard, in the current study about half (51%) of the participants mentioned being amenorrhic as a reason for not using contraceptive.

Limitations of the study are it mainly focuses on individual level factors and factors related to the health system and the service providers did not included, the socio cultural factors and related misconception on family planning did not assessed and women who were not using contraceptives, but were abstaining from sexual intercourse, were not considered to be protected from unintended pregnancy. Since it is cross-sectional study cause effect relation is not established.

## Conclusion

In this study, about half of the study subjects were not using modern contraception despite their need to limit their family size or space next pregnancy. This is higher than unmet need of family planning rate among married women of reproductive age group signifies that women in the extended post-partum period had less intention to use modern contraceptives. This study also demonstrated that the majority of women in the extended post-partum period were using short term hormonal family planning methods (mainly Injectable) than the long term methods.

Illiteracy, non-use of ANC service, non-use of PNC service and being knowledgeable about LAM were the main predictors that increase likely hood of unmet need in the extended postpartum period.

Based on the findings the following recommendations were given. As maternal health services (ANC, institutional delivery and PNC) are potential times for counseling of mothers about post-partum FP, counseling about modern family planning should get more focus. There is great need to prioritize education of girls to empower them to use postpartum FP and policies need to encourage women to be supported by their spouses and to promote postpartum family planning. In addition there is need to improve the quality of knowledge on the recommended postpartum contraceptives to enable extended post-partum mothers make informed choices, which can be facilitated by strengthening client-provider interaction especially through maternal and child health services.

## Abbreviations

ANC: Antenatal Care
AOR: Adjusted Odds Ratio
CI: Confidence Interval
COR: Crude Odds Ratio
DHS: Demographic and Health Survey
EDHS: Ethiopia Demographic and Health Survey
FP: Family Planning
IUCD: Intrauterine Contraceptive Device
LAM: Lactational Amenorrhea Method
PNC: Postnatal Care
